# Epidemiological characteristics of spinal cord injury in Northwest China: a single hospital-based study

**DOI:** 10.1186/s13018-020-01729-z

**Published:** 2020-06-09

**Authors:** Zhi-Meng Wang, Peng Zou, Jun-Song Yang, Ting-Ting Liu, Lei-Lei Song, Yao Lu, Hao Guo, Yuan-Ting Zhao, Tuan-Jiang Liu, Ding-Jun Hao

**Affiliations:** 1 Department of Spinal Surgery, Honghui Hospital, Xi’an Jiaotong University, No.76, Nanguo Road, Beilin District, Xi’an, 710054 Shaanxi China; 2 Xi’an Medical University, Xi’an, Shaanxi China; 3 Department of Orthopaedics and Trauma, Honghui Hospital, Xi’an Jiaotong University, No.76, Nanguo Road, Beilin District, Xi’an, 710054 Shaanxi China; 4 Department of Pediatric, Northwest Women’s and Children’s Hospital, Xi’an, Shaanxi China; 5 Qinghai University, Xi’ning, Qinghai China

**Keywords:** Spinal injuries, Epidemiology, Northwest China, Retrospective study, Investigation

## Abstract

**Background:**

While the cities in China in which spinal cord injury (SCI) studies have been conducted previously are at the forefront of medical care, northwest China is relatively underdeveloped economically, and the epidemiological characteristics of SCI have rarely been reported in this region.

**Methods:**

The SCI epidemiological survey software developed was used to analyze the data of patients treated with SCI from 2014 to 2018. The sociodemographic characteristics of patients, including name, age, sex, and occupation, were recorded. The following medical record data, obtained from physical and radiographic examinations, were included in the study: data on the cause of injury, fracture location, associated injuries, and level of injury. Neurological function was evaluated using the American Spinal Injury Association (ASIA) impairment scale. In addition, the treatment and complications during hospitalization were documented.

**Results:**

A total of 3487 patients with SCI with a mean age of 39.5 ± 11.2 years were identified in this study, and the male to female ratio was 2.57:1. The primary cause of SCI was falls (low falls 47.75%, high falls 37.31%), followed by traffic accidents (8.98%), and impact with falling objects (4.39%). Of all patients, 1786 patients (51.22%) had complications and other injuries. According to the ASIA impairment scale, the numbers of grade A, B, C, and D injuries were 747 (21.42%), 688 (19.73%), 618 (17.72%), and 1434 (41.12%), respectively. During the hospitalization period, a total of 1341 patients experienced complications, with a percentage of 38.46%. Among all complications, pulmonary infection was the most common (437, 32.59%), followed by hyponatremia (326, 24.31%), bedsores (219, 16.33%), urinary tract infection (168, 12.53%), deep venous thrombosis (157, 11.71%), and others (34, 2.53%). Notably, among 3487 patients with SCI, only 528 patients (15.14%) received long-term rehabilitation treatment.

**Conclusion:**

The incidence of SCI in northwest China was on the rise with higher proportion in males; fall and the MCVs were the primary causes of SCI. The occupations most threatened by SCI are farmers and workers. The investigation and analysis of the epidemiological characteristics of SCI in respiratory complications are important factors leading to death after SCI, especially when the SCI occurs in the cervical spinal cord. Finally, the significance of SCI rehabilitation should be addressed.

## Background

Spinal cord injury (SCI) contributes to serious functional and financial burden and poses a series of problems for the patient’s mental health and social stability [1]. SCI is considered to be a major public health problem worldwide, and the incidence of SCI varies greatly between regions [2]. The average annual incidence of SCI in developed countries ranges from 10.4 per million persons to 83 per million persons [3, 4]. In developing countries, SCI has a high incidence of 25.5 per million persons per year [5]. Considering the lack of effective rehabilitation methods for SCI, primary prevention is particularly important.

A few studies have reported the epidemiological characteristics of SCI based on data from hospitals in different parts of China, including Beijing [6], Shanghai [7], Guangdong [8], and Chongqing [9]. In terms of the epidemiology of SCI, the characteristics and occurrence vary greatly; thus, it is important to conduct epidemiological studies on SCI at the population level. While the cities in China in which SCI studies have been conducted previously are at the forefront of medical care, northwest China is relatively underdeveloped economically, and the epidemiological characteristics of SCI have rarely been reported in this region. Hence, this study aims to investigate the epidemiological characteristics of SCI in northwest China, to facilitate optimal medical resource allocation for reducing the financial and social burden of SCI.

## Materials and methods

This study was approved by the Ethics Committee of our hospital. As the hospital is the tertiary trauma center in northwest China, we were able to obtain a large sample size of SCI patients from this hospital. Patients with traumatic SCIs or cauda equina injuries who were admitted to the hospital between 2014 and 2018 were included in the study, while patients who met the following criteria were excluded: (1) vertebral body fractures without SCI, (2) neurological deficit caused by degenerative spinal disease, (3) fatal injuries, and (4) incomplete medical records. The SCI epidemiological survey software developed was used to analyze patient data. The sociodemographic characteristics of patients, including name, age, sex, and occupation, were recorded. The following medical record data, obtained from physical and radiographic examinations, were included in the study: data on the cause of injury, fracture location, associated injuries, and level of injury. Neurological function was evaluated using the American Spinal Injury Association (ASIA) impairment scale. In addition, the treatment and complications during hospitalization were documented.

### Statistical analysis

Mean values are presented as the mean ± standard deviation (SD). The analysis of variance (ANOVA) and chi-squared tests were used to analyze continuous and categorical data, respectively. A value of *p* < 0.05 was considered statistically significant. All statistical analyses were performed using Statistical Product and Service Solution Version 19.0 (SPSS, Inc., Chicago, IL, USA).

## Results

### General demographic characteristics of SCI patients between 2014 and 2018

**Fig. 1:**
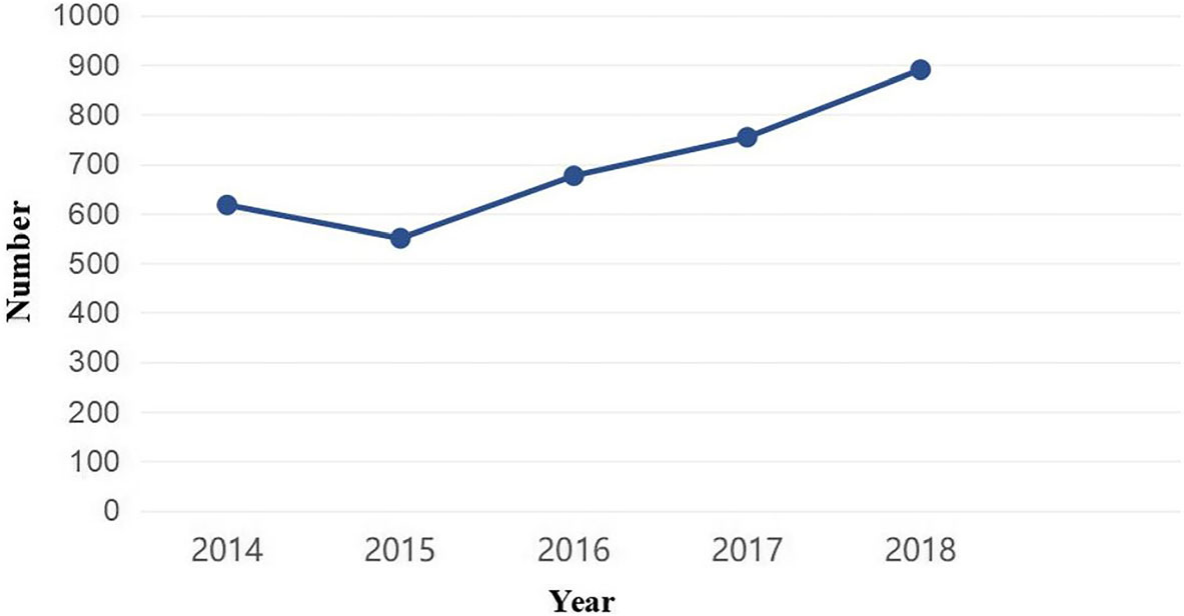
The trend of SCI patients during 2014–2018

A total of 3487 patients with SCI were identified in this study (Fig. 1). Table 1 shows the general demographic characteristics of SCI patients. Of the 3487 individuals with SCI, 2509 were male (71.95%) and 978 (28.05%) were female; the male to female ratio was 2.57:1. Patient age ranged from 18 to 87 years, with a mean age of 39.5 ± 11.2 years (male, 36.6 ± 12.4 years; female, 42.8 ± 11.8 years). The proportions of farmers and workers were as high as 59.51% and 27.04%, respectively.

**Table 1:** Demographic information, etiology of patients with SCI from 2014 to 2018

Years	2014	2015	2016	2017	2018	Total
Age						
0–20	2	0	1	1	5	9
20–29	86	79	93	117	146	521
30–39	335	274	361	344	385	1699
40–49	114	107	143	198	228	790
50–59	67	85	70	83	109	414
≥ 60	13	5	8	11	17	54
Gender						
Male	446	413	504	533	613	2509
Female	171	137	172	221	277	978
Occupation						
Farmer	387	354	401	442	491	2075
Worker	155	138	179	213	258	943
Government-offices	43	37	58	61	68	267
Retired	8	6	8	15	36	73
Students	15	10	18	13	19	75
Other*	9	5	12	10	18	54
Etiology						
Low fall	301	277	328	358	401	1665
High fall	252	223	241	259	326	1301
MVCs	39	41	72	77	84	313
Fall objects	15	4	24	47	63	153
Sports	8	4	6	9	13	40
Violence	2	1	5	4	3	15
Total number	617	550	676	754	890	3487

Other* included unemployed individuals and self-employed individuals

*MVCs* motor vehicle collisions

### Etiology of injury and age distribution of patients with SCI

In this study, the primary cause of SCI was falls (low falls 47.75%, high falls 37.31%), followed by traffic accidents (8.98%), and impact with falling objects (4.39%). Furthermore, several unusual causes of SCI, such as those involving sports injuries (1.15%) and violence injuries (0.43%), were also reported. The peak age of patients with SCI ranged from 30 to 49 years, and these patients accounted for 80.99% of all patients. Further, SCI incidence was negatively correlated with age. The etiologies of injuries among different age groups are shown in Table 2. The common etiologies in the 30–39 years age group were falls (low and high falls) and motor vehicle collisions (MVCs). Low falls were the primary cause of SCI in patients in the 60-year-old age group, while low falls and MVCs were the primary causes of SCIs in patients aged between 20 and 29 years.

**Table 2:** Analysis of the etiologies and age distribution among the spinal cord injury (SCI) patients

Etiologies	Age						Total
	0–20	20–29	30–39	40–49	50–59	≥ 60	
Low fall	0	213	733	420	262	37	1665
High fall	1	199	759	282	53	7	1301
MVCs	4	75	132	58	34	10	313
Falling objects	0	9	61	21	62	0	153
Sports	3	21	10	6	0	0	40
Violence	1	4	4	3	3	0	15

*MVCs* motor vehicle collisions

### Level of injury and associated injuries

**Fig. 2:**
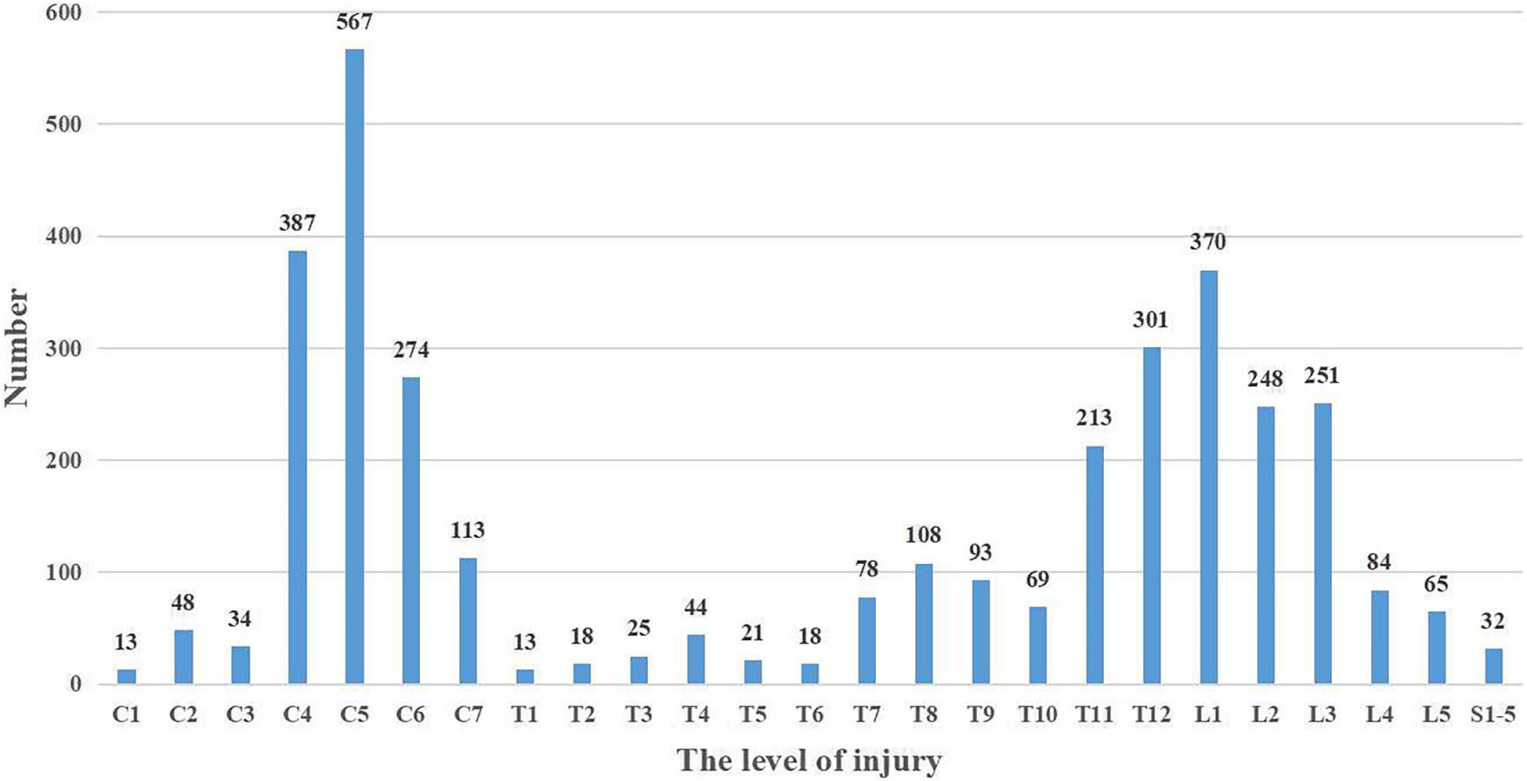
Distribution of spine level injuries for SCI patients by the severity of injury

As shown in Fig. 2, an analysis of fracture locations revealed a bimodal distribution. The first peak was seen for the cervical region (41.2%), especially C4-C6, with the second peak observed for the thoracolumbar region (25.3%). Of all patients, 1786 patients (51.22%) had complications and other injuries, including craniocerebral injury (198, 11.09%), frontofacial injury (407, 22.79 %), chest and abdominal injuries (359, 20.10%), pelvic injury (258, 14.45%), and limb fracture (564, 31.58%).

### ASIA impairment scale

According to the ASIA impairment scale, the numbers of grade A, B, C, and D injuries were 747 (21.42%), 688 (19.73%), 618 (17.72%), and 1434 (41.12%), respectively. As shown in Table 3, the different severities of SCI injury were related to different causes: MVCs and high falls, grade A injuries and low falls, grades B and C injuries. Further, different sites of injury were related to different grades of injuries. Injuries to the cervical cord and lumbar cord widely corresponded to ASIA grades A and D, while injuries to the thoracic cord were mostly associated with ASIA grades A and B. Moreover, sacral cord injuries were mainly related to ASIA grade D, as shown in Table 4.

**Table 3:** Comparison of causes of injury in different degrees of spinal cord injury

Etiologies	ASIA scale			
	A (%)	B (%)	C (%)	D (%)
Low fall	129 (17.27)	267 (38.81)	293 (47.41)	976 (68.06)
High fall	410 (54.89)	277 (37.08)	249 (40.29)	365 (25.45)
MVCs	136 (18.21)	78 (11.34)	51 (8.25)	48 (3.35)
Falling objects	57 (7.63)	58 (8.43)	14 (2.27)	24 (1.67)
Sports	8 (1.07)	5 (7.27)	7 (1.13)	20 (1.39)
Violence	7 (0.94)	3 (4.36)	4 (0.65)	1 (0.07)

**Table 4:** Analysis of the degrees and segment of the injury among the SCI patients

ASIA scale	The level of injury			
	Cervical cord (%)	Thoracic cord (%)	Lumbar cord (%)	Sacral cord (%)
A	516 (31.29)	267 (30.20)	96 (10.29)	0 (0.00)
B	249 (15.10)	277 (31.33)	198 (21.22)	0 (0.00)
C	121 (7.33)	78 (8.82)	167 (17.90)	0 (0.00)
D	763 (46.27)	0 (0.00)	472 (50.59)	21 (100)

### Treatment of SCI and clinical complications

Of all patients, 2763 (79.24%) received surgical treatment and 724 (20.76%) received conservative treatment. The duration of hospitalization of patients with SCI ranged from 1 to 378 days, with an average of 17.50 days. During the hospitalization period, a total of 1341 patients experienced complications, with a percentage of 38.46% (Table 5). Among all complications, pulmonary infection was the most common (437, 32.59%), followed by hyponatremia (326, 24.31%), bedsores (219, 16.33%), urinary tract infection (168, 12.53%), deep venous thrombosis (157, 11.71%), and others (34, 2.53%). Notably, among 3487 patients with SCI, only 528 patients (15.14%) received long-term rehabilitation treatment.

**Table 5:** Clinical complications during the hospitalization

Complication	Number (%)
Pulmonary infection	437 (32.59%)
Hyponatremia	326 (24.31)
Bedsore	219 (16.33)
Urinary tract infection	168 (12.53)
Deep venous thrombosis	157 (11.71)
Others^#^	34 (2.53)

Others^#^ include cardiovascular diseases and digestive system disease

## Discussion

A recent systematic review that included 17 studies in China showed that the epidemiological features of SCI vary among different regions of China; therefore, targeted prevention interventions should be implemented by region. Further, SCI resulting from falls and MVCs may become a major public health concern as the population ages and the economy continues to develop in China [10]. Compared to other areas of China, northwest China has several unique characteristics. First, it is located in the hinterland of mainland China, which mostly consists of plateaus and basins. Compared with the economically developed eastern coastal areas, the level of economic and political development is low in this region. In addition, northwest China has a landscape dominated by agriculture and farmers account for the majority of the labor force; low levels of health insurance coverage, education, and household income are also observed in this region. As Xi’an is the economic and cultural center of northwest China, the epidemiological characteristics of SCI patients admitted to the tertiary trauma center in Xi’an are representative of those of SCI patients in northwest China. Based on the epidemiological characteristics of SCI over the past 5 years, we found that SCIs not only cause the impairment of sensory and motor functions below the injury level but also cause several debilitating organ dysfunctions, including those of the respiratory, urinary, and digestive systems, which burdens hospitals with additional costs. Hence, SCIs should not be neglected in northwest China. As the prevention of SCIs is particularly important, comprehensive and detailed epidemiological investigation is fundamental for the development of effective prevention countermeasures.

In this investigation, the male to female ratio in SCI patients was 2.57:1, which was different from the ratios reported in Beijing, Shanghai, Guangdong, Chongqing, Anhui, and Heilongjiang [6–9, 11, 12]. This may be due to the differences in responsibilities and social division of labor between men and women among various provinces of China. Our patients mainly came from the Northwest region, which is economically underdeveloped and resource-poor. The exposure of women to high-risk industries, such as construction and transportation, has been on the rise. Simultaneously, women are prone to osteoporotic fracture, which may result in higher proportions of women among SCI patients; this has been previously observed in South Africa [13].

The highest proportion of SCIs in northwest China was noted among patients aged between 30 and 49 years. In traditional Chinese culture, it is the responsibility of the young and middle-aged individuals to support their parents and raise their children. Thus, due to the large financial responsibility, they take great risks to provide for their families. Additionally, the roads in northwest China are rugged and undeveloped. Hence, these factors increase the possibility that young and middle-aged individuals experience work-related SCIs, rendering the 30–49 years age group a high-risk group. Moreover, as China’s aging population is increasing, more elderly people experience SCIs. These patients may have comorbidities such as degenerative spine disease and/or osteoporotic compression fractures. Therefore, the needs of elderly people should be considered in SCI rehabilitation [14]. In our study, the proportions of farmers and workers were as high as 59.51% and 27.04%, respectively. These values are different from those reported in previous studies conducted in the Guangdong region of China [8], Turkey [15], and Mexico [16]. These discrepancies may be a result of the differences in economic and political environments between regions. In northwest China, a high proportion of the population engages in agriculture-related occupations, which are related to a higher probability of SCI occurrence than any other occupation.

The causes of SCIs include falls (high and low falls), MVCs, impact with falling objects, sports, and violence injuries, and these causes vary across countries and regions. An epidemiological survey conducted in Canada in 2006 showed that MVCs were the main cause of SCIs, while falls became the main cause in 2009 [17, 18]. Another study from seven countries in the Middle-East and North Africa (MENA) region found that MVCs are still the leading cause of SCIs, followed by falls, gunshots, violence, and sports [19]. We found that falls (both from a small and large height) and MVCs were the main causes of SCIs and occurred in nearly all age groups. The incidence of violence also varied by country and region, with the incidence of SCI due to violence being as low as 0.40% in Beijing [6] and as high as 28.4% in Brazil [20]. In the same fashion, gunshot wounds were rare in China, mainly due to the strict social security and gun control implemented by the state. As in other developing countries, the per capita car ownership in China is increasing; meanwhile, the improvement in transportation safety measures and the increase in traffic safety awareness have resulted in a decline in traffic-related SCIs.

Similar to the findings of previous studies [9, 21], the analysis of injury locations in this study showed a bimodal distribution, with C4-C6 and T11-L1 being the most common locations of injury. Additionally, we found an association between the severity of SCI and the cause of injury. While injuries resulting from MVCs and falls from a large height mostly lead to complete SCIs, mainly of grade A, falls from a small height primarily cause grade D SCIs (incomplete SCI). Williams et al. [22] and Thietje et al. [23] reported that patients with grade A SCIs are more likely to experience depressive disorders and suicide; therefore, the families and doctors of these patients should provide more care to these patients to help prevent suicide caused by depression.

The results of this study showed that there were 1341 (36.49%) patients with complications, with respiratory disease being the most common complication (30.7%). Respiratory disease is associated with long-term bed rest, lung disease caused by smoking, and rib fractures. Cervical SCIs may affect the function of the diaphragm or intercostal muscle, weaken respiration, and cause coughing, making it difficult to cough out sputum. Such symptoms may also present as complications related to respiratory disease [24]. The higher is the level of SCI, the higher is the risk of pulmonary infection. The risk of pulmonary infection can reach up to > 90% when the SCI occurs above the C5 level, causing dysfunction of the diaphragm [25]. Our results also showed that the average hospitalization duration of SCI patients was 10.70 days, with the longest stay being 94 days, while the hospitalization cost was between 4352 and 456,320 yuan (average 37,850 yuan). It has been highlighted that the hospitalization period of SCI patients is long, the hospitalization cost is high, and the patients have a limited ability to pay for treatment as their income is low; hence, it is difficult to provide comprehensive and effective treatment.

Of all SCI patients, only 15.14% received rehabilitation. Although this may be related to the low overall rate of SCI rehabilitation in northwest China, it reflects the insufficient attention given to SCI postoperative rehabilitation.

The study has several limitations. First, it was a hospital-based descriptive study on SCI that identified only a small proportion of all SCI patients in northwest China. Second, we collected information of patients admitted to the hospital with SCI, leaving out the information on patients who died in hospitals before admission. Third, training on systematic data collection was not provided, resulting in errors in the data collection process.

## Conclusions

The investigation and analysis of the epidemiological characteristics of SCI in northwest China suggest the requirement of further research on the epidemiology of SCI in this region. Additionally, education regarding the safety and protection of high-risk groups should be strengthened to reduce the incidence of catastrophic SCIs. Moreover, our study showed that respiratory complications are important factors leading to death after SCI, especially when the SCI occurs in the cervical spinal cord. Finally, the significance of SCI rehabilitation should be addressed.

## Data Availability

The datasets generated during the current study are public at the email dingjun.hao@qq.com.

## Abbreviations

ANOVA: Analysis of variance
ASIA: American Spinal Injury Association
MENA: Middle-East and North Africa
SCI: Spinal cord injury
SD: Standard deviation
